# Cancer Patients with Chronic Pain and Their Caregivers during COVID-19: A Descriptive Study

**DOI:** 10.3390/nursrep13030082

**Published:** 2023-06-26

**Authors:** Cristina Costeira, Filipe Paiva-Santos, Nelson Pais, Ana Filipa Sousa, Ivo Paiva, Dulce Helena Carvalho, Ana Rocha, Filipa Ventura

**Affiliations:** 1 ciTechCare, Rua de Santo André–66-68, Campus 5, Polytechnic of Leiria, 2410-541 Leiria, Portugal; 2 Health Sciences Research Unit: Nursing (UICISA: E), Nursing School of Coimbra (ESEnfC), 3004-011 Coimbra, Portugal; filipesantos@esenfc.pt (F.P.-S.); 3744@ipocoimbra.min-saude.pt (A.F.S.); filipaventura@esenfc.pt (F.V.); 3 School of Health Sciences of Polytechnic of Leiria, Campus 2, Morro do Lena, Alto do Vieiro, Apartado 4137, 2411-901 Leiria, Portugal; 4 Portuguese Oncology Institute of Coimbra, 3004-011 Coimbra, Portugal; 1486@ipocoimbra.min-saude.pt (N.P.); ivopaiva3@esenfc.pt (I.P.); 660@ipocoimbra.min-saude.pt (D.H.C.); 2112@ipocoimbra.min-saude.pt (A.R.)

**Keywords:** COVID-19, chronic pain, patient, caregivers, oncology, anxiety, depression, stress

## Abstract

Background: The sanitary measures imposed by COVID-19 intensified challenges in the pain management of cancer patients. Methods: A descriptive study was conducted in a chronic pain unit of an oncological hospital aiming to explore the experiences of cancer patients with chronic pain and their caregivers during the pandemic period, as well as identify strategies to improve care in chronic pain management. An electronic questionnaire was developed containing sociodemographic variables, the Depression, Anxiety and Stress Scale-21, and open-ended questions exploring the experiences and circumstances of pain management. Results: A total of 30 patients and 13 caregivers filled in the questionnaire. Patients revealed a higher level of depression, anxiety, and stress than caregivers, without statistically significant differences. Both groups mentioned having experienced difficulties in self-care, particularly in relation to sleep, nutrition, and recreation. In total, 83.7% patients needed pain relief medication related to uncontrolled pain. Both mentioned that they would have benefited from a digital application to ease the communication with the healthcare professionals of the chronic pain unit, as well as non-pharmacological interventions, such as therapeutic massage. Conclusions: Recognizing that chronic pain leads to significant limitations, it is essential to implement and anticipate objective and effective responses in pain management.

## 1. Introduction

Pain is a common symptom in cancer patients, with 70% of patients being expected to report it [1]. Based on its duration, pain can be categorized as acute or chronic, with chronic pain lasting longer than three months [2]. Chronic pain in particular affects 40–70% of the patients with a cancer diagnosis and approximately 32% of these cases are estimated to be undertreated, which compromises the Quality of Life (QoL) of both patients and their caregivers [3,4,5,6,7,8]. Additionally, pain leads to an increase in medical expenditures and significant economic costs [9].

To reduce the complications associated with chronic pain and improve the quality of care, healthcare professionals need to be aware of and implement strategies to an effective chronic pain management for both patients and their caregivers in a continuous manner throughout the clinical pathway [10,11].

Therefore, effective chronic pain management is crucial in many domains both at the patient- and the organizational-level and should be integrated in a comprehensive assessment and symptom control performed by the healthcare professionals [1,12,13,14]. To achieve an optimal pain management for cancer patients, standard care recommends the combination of pharmacological measures (e.g., opioid medication) with physical therapy (e.g., exercise, acupuncture, massage, and transcutaneous electric nerve stimulation), psychosocial therapy (e.g., mindfulness, supportive therapy), and herbal supplementation [12,15,16,17].

### 1.1. Theoretical Framework

The biopsychosocial model of pain is the golden standard in the understanding of chronic pain among the scientific community [18]. This framework presents a dynamic interaction between biological, psychological, and social factors that shape an individual’s response to chronic pain [18,19,20].

When uncontrolled, chronic pain often negatively impacts the person’s lifestyle and daily activities, such as sleep, diet, physical activity, consumption habits, demanding a substantial effort from the patients and caregivers to regain control [21].

In a context of chronic pain experience, the patients are challenged to develop a set of new behaviors to promote symptomatic control and enhance QoL. Such an approach benefits from the support of informal caregivers, who may support the daily activities and restore autonomy and QoL [22].

Self-care is as an important area of nursing practice, since it has a significant impact on the way in which the person manages chronic disease and the inherent therapeutic regimen [19,23].

The consecutive decrease in QoL, the chronic pain and its impact in the self-care ability additionally bring uncertainty, as the structure of daily life is disrupted and the person must readjust their routines and activities. Living with chronic pain requires an effective adaptation to a health-disease transition. Specifically, a poorly coordinated transition can potentiate the risk of re-hospitalization or even death [24,25].

### 1.2. Research Problem

COVID-19 has affected the self-management of patients with chronic disease all over the world [26], raising new challenges for patients, caregivers, and healthcare professionals and requiring a rapid adaptation to the new reality [27]. Patients and caregivers experienced significant consequences in anticipatory grief, leading to a high degree of suffering, a decrease in QoL, and consequent psychological morbidities [28].

The sanitary measures to control the COVID-19 dissemination led to a sustained reduction in the number of cancer patients diagnosed, referred, and treated [29], and altogether to a worst chronic pain assessment and control than before [14].

The recent evidence emphasizes the importance of considering a dyadic perspective (patient–caregiver) to the understanding of the pain experience and of its impact on the QoL of both patients and caregivers [5].

### 1.3. Objectives

The present study aimed to explore the experiences of cancer patients with chronic pain and their caregivers during the COVID-19 pandemic period, as well as identify strategies to improve care in chronic pain management.

## 2. Materials and Methods

### 2.1. Study Design

A descriptive study was developed with cancer patients with chronic pain and their caregivers. It was used the STROBE Statement—checklist of items that should be included in reports of observational studies (Table A1).

### 2.2. Setting

The study was carried out during the COVID-19 pandemic period, in a pain unit of a hospital in the center region of Portugal, during 2021 until March 2022. The pain unit is manned by a multidisciplinary team composed of two nurses, five physicians with pain management expertise recognized from physicians’ professional order, a social worker, and a psychologist. During the study’s period, the healthcare team followed 362 new cancer patients with chronic pain, in a total of 1758 appointments, which was about 20% less compared to the same period before the COVID-19 disease.

The pain unit offers pharmacological and non-pharmacological approaches to pain management in a complementary relationship. Concerning the non-pharmacological approaches, the oncology nurses working at the unit provide a systematic program composed of therapeutic massage and relaxation techniques, aiming for efficient pain management. The program takes place once a week over the course of eight weeks; however, it was interrupted during the research period due to the sanitary measures imposed.

### 2.3. Participants

Convenience sampling was used to recruit participants to this study. The participation was based on a voluntary decision, after the individual made the informed decision to participate. In this study, we included all patients who had a planned appointment at the pain unit during the study period and their informal caregivers, and who consented to participate after invitation from the healthcare team. The invitation was made by one of the nurses from the pain unit team in the waiting room, during the time when patients and caregivers were expecting their planned appointment.

#### 2.3.1. Inclusion Criteria

The inclusion criteria for the patients’ sample were having a cancer diagnosis, regardless of the disease or the treatment stage; having a chronic pain diagnosis; and having an appointment with the healthcare team at the pain unit during the study period. All informal caregivers accompanying the patients to the pain unit appointment were invited to participate in the study. Patients and informal caregivers should preferably have digital skills to complete data collection. The digital skills were tested by nurses through a test survey applied to the participants, before providing the final link to the questionnaire. All invited people were required to consent to participation.

#### 2.3.2. Exclusion Criteria

All patients and informal caregivers who were not able to complete the questionnaire were excluded from the study. The responses of participants who decided to withdraw from the study were also excluded.

### 2.4. Data Collection

An online questionnaire was made available on a tablet by the pain unit nurse or sent by email according to the participants preference. The questionnaire was estimated to take around 10 min to be completed.

The online questionnaire was composed of sociodemographic variables such as sex, age, marital status, education level, and residential area. The second part included the Depression, Anxiety and Stress Scale-21 (DASS-21) [30] and the third part was composed of open-ended questions on possible strategies that could have been facilitators of pain management and control, as well as participants experiences during the pandemic period. Previously to the beginning data collection start, the third part of the questionnaire was tested with 20 patients to assess its readability and content adequacy.

DASS-21 enables the assessment of the levels of depression, anxiety, and stress. Each dimension is composed of seven questions, answered on a 5-point Likert scale, ranging from totally disagree (1) to totally agree (5), with higher levels indicating a greater level of the assessed construct. The scale has been translated and culturally adapted to Portuguese and revealed evidence of validity and good reliability in a sample of 200 subjects by Ribeiro-Pais et al. [30]. In the current study it showed excellent reliability in both groups and across dimensions [31]. The total Cronbach alpha were 0.94 for the patient’s group and 0.95 to the caregivers group.

### 2.5. Data Analysis

Descriptive and inferential statistics were used. Frequencies, percentages, medians, means, and standard deviations were determined for sample description. Since the data were not normally distributed, non-parametric tests were performed. Mann–Whitney U was used to compare differences between two independent groups, i.e., patients and caregivers. Quantitative data were analyzed using SPSS-26 at a significance level of 0.05. To analyze the data obtained from open-end question the maxQDA^®^ software version 2022.6, was used aiming for a content analysis.

### 2.6. Ethical Considerations

The study was reviewed and approved by the Oncology Hospital’s Ethical Committee (TI no 20/2020). Verbal and written information about the study was provided to all the participants. Responses were anonymous and the self-determination principle was guaranteed. The participants gave their agreement to participate in the study before starting the online questionnaire.

## 3. Results

### 3.1. Sample Characteristics

A total of 43 participants were involved in the study, including 30 patients and 13 informal caregivers. Most participants were women (*n* = 31). The mean age of the patients was 55.47 ± 09.67 years (range 36–75), and for the informal caregivers the mean age was 51.54 ± 09.24 years (range 41–65). All the informal caregivers were married and had higher-school education (53.9%). As for the cancer patients, most of the participants had a basic education (76.7%).

Most of participants recognized the importance of a digital application to strengthen the communication between patients and caregivers and healthcare professionals, such as mobile Apps. Maintaining the massage therapy and relaxation sessions during the confinement period was understood as an aspect that could not to be neglected and should, therefore, not be canceled, since it was identified as an important chronic pain management strategy (Table 1).

All in all, 46.7% were in need of pain relief medication, which might not have been enough since the majority (86.7%) said that they sought further support from the pain unit.

For the first open-ended question, the answers revealed four categories of facilitating strategies for pain management and control during the confinement period, including frequent telephone calls, videoconferences, physical approaches, and psychosocial approaches.

From the perspectives of both the patients and the caregivers, the use of telecommunication during the pandemic time to monitor and follow-up was an important strategy, corroborated by an increase in the number of regular phone calls (100%) and video-conference services (≈40%). Other non-pharmacologic approaches were also valued, such as physical (e.g., massage “I would like to have my acupuncture sessions to control my pain…” and psychosocial (e.g., supportive therapy: “P7: “talk with Dr. xxx [psicologist]…).

Concerning the lived experiences, the results evidence two categories, including changes in the self-care activities and personal suffering. All the participants mentioned interferences on their self-care activities, particularly portrayed in inadequate nutrition (C4: “*I didn’t have enough food at home, and I didn’t had way to go out to buy it, because I was afraid to catch COVID and transmit it to my husband*…” and absence of leisure activities (P21: “*I missed my card game*…”.

Personal suffering was portrayed on descriptions of despair (P3 “*I was scared and terrified with the situation, I was so afraid that I would never see my family again, and day after day I was losing all hope in future and in the possibility to survive*…”.

**Figure 1:**
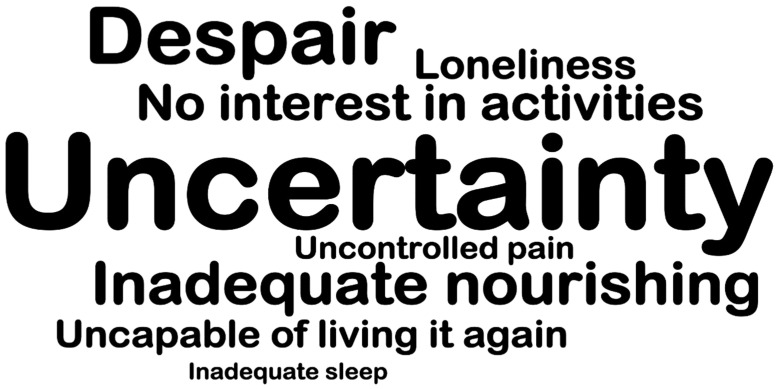
Pandemic experiences.

Figure 1 depicts a wordcloud delivered from the qualitative analysis software, which shows the words most frequently written, in open-end questions, by the participants. The uncertainty and despair were the most frequent words written.

### 3.2. Depression, Anxiety and Stress Assessment

Table 2 presents the assessment results for depression, anxiety, and stress by the DASS-21 for both patients and informal caregivers.

The results suggest that both patients and caregivers had similar outcomes for depression, anxiety, and stress. Patients revealed a higher level of depression, anxiety, and stress than caregivers. Despite these outcomes, no statistically significant differences were found between the two groups.

## 4. Discussion

Cancer patients with chronic pain and their informal caregivers experienced difficult times during the confinement period. The changes in daily life impacted on self-care and increased personal suffering. This reality has been described in other studies [32,33,34], that show that cancer patients were advised to stay at home as they were considered at high-risk of developing the infection of COVID-19. To further reduce the risk of contamination, the informal caregivers of cancer patients were also advised to remain in confinement with their family members, which consequently led to the interruption of their own personal and clinical routines [32,33,34]. This evidence might explain the results of the current study concerning self-care activities and personal suffering. Specifically, the loneliness experienced by informal caregivers, even though they were not alone at home, which should constitute a focus of attention of the healthcare team. In particular, the informal caregivers also mentioned experiencing pain, as they were also taking pain relief medication in the current study.

The informal caregivers are essential partners in caring for the person with cancer. Ensuring their well-being is as important as caring for the patient with cancer, who might see the care and support at home jeopardized otherwise [35,36], with a consequently unsuccessful pain management. Importantly not all patients complied with the prescription of medication for pain control, which additionally compromises effective chronic pain management.

The pandemic confinement period brought countless difficulties and constraints to patients and caregivers. The postponed or canceled follow-up pain appointments and treatments led to dissatisfaction and difficulties in the health/illness management experienced by patients and their caregivers [32]. In the current study, most of the patients and caregivers considered they had received adequate follow-up from healthcare professionals in general and from the pain unit, with whom the majority-maintained contact. Yet, the results portray a negative impact on mental wellbeing and not all of them completed their massage therapy according to plan. Specifically, the levels of depression, anxiety, and stress were found to be moderate in both groups in the current study. These results are aligned with other studies [37], corroborating that COVID-19 pandemic isolation measures were responsible for limiting the access to non-pharmacologic therapies, such as massage and supportive therapy, and exacerbated mental health disorders, including anxiety and depression [14].

It is noteworthy that the results showed a negative impact on the sleep and nutrition patterns, as well as recreation, reinforcing the need to conduct a holistic assessment of the person in his/her context for optimal pain management.

In this study, the patients and informal caregivers identify telehealth in the form of frequent phone calls and videoconference, as an important tool that they would have liked to have more often during the confinement period. The remote interactions were adopted worldwide during the pandemic and shifted the paradigm for chronic pain care [38,39]. Telemedicine brings new opportunities for providing valuable care for patients with significant barriers to access healthcare professionals [39]. Telemedicine may provide equitable, accessible, and affordable healthcare to patients and caregivers [40] and is an essential tool that should be improved and embedded, even in regular practice.

The participants in this study identified the ease of contact with the pain unit team for emergent issues, as well as for regular follow-up as an important asset and essential to pain self-management at home, generally welcoming the use of an App and telehealth, including video and sound, to facilitate communication in situations where face-to-face contact was not advised or was compromised. Importantly, most of the participants in both groups identified the rural area as their area of residence. The acceptability and effectiveness of eHealth tools to complement communication and supportive processes throughout the cancer care journey are increasingly recognized [33], particularly to bridge geographical disparities [41]. Yet, evidence suggests the need for embedding such innovative tools as part of a clinical workflow, ideally grounded on person-centered care principles so that they are put in use to favor the therapeutical relationship and shared decision-making at distance [42,43].

Recent evidence, aligned with calamity situations, recommends a comprehensive and systematized resilience assessment from the early stage of the health/illness journey in order to provide personalized support and interventions tailored to answer to the specific needs of individuals. Such assessment demands organizational changes, and the application of new technologies in pain medicine, especially when dealing with vulnerable groups [44,45].

Concerning the non-pharmacologic therapies, massage and supportive therapy were strategies identified as important by patients undergoing pain treatment. In this regard, the non-pharmacologic approaches should also be discussed before deciding about its suspension or delay.

Overall, the results of the current study shed light on the need to attend to the dyad patient-caregiver throughout the care pathway. This evidence is not new and is particularly developed in the domain of palliative care [46]. The results of this study demand a reflection from oncology healthcare professionals concerning the optimal strategies to care for both the patient and the caregiver, particularly in situations where the face-to-face contact is compromised. Further research should explore the transferability of interventions to attend to the dyad patient-caregiver from the early stage of the health/illness journey in the oncological care pathway, especially aiming for the promotion of wellbeing through non-pharmacological interventions. Here, oncology nurses play a crucial role in the dyad needs’ assessment and in the implementation of interventions [47].

As a study limitation, the sample size should be taken into consideration when seeking transferability of results across contexts. Having digital skills as an inclusion criterium might be considered to have biased the results. Future studies should consider alternative options to data collection that do not demand digital skills or provide assistance in digitally supported data collection.

## 5. Conclusions

These contributions are expected to help healthcare professionals to rethink healthcare pathways in future similar situations.

Person-centered care is a fundamental requirement in cancer patients with chronic pain and should attend to the needs of both patients and their caregivers in maintaining wellbeing. The oncology healthcare team’s role further encompasses the need for an effective follow-up to the dyad patients-caregivers, which is of particular significance in chronic pain management.

Recognizing that chronic pain leads to significant impairment and changes in daily activities, the anticipation, planning, and implementation of effective responses to pain management is essential. Here, the intermittent face-to-face contact inherent to the outpatient oncology pathway should be redesigned, so to attend to the continuous of the pain experience, its assessment and implementation of non-pharmacological interventions, if aiming to the prevention of major damage.

## Figures and Tables

**Table 1:** Demographic characteristics of participants (*n* = 43), circumstances and opinions.

	Patients, *n* = 30 *n* (%)	Caregivers, *n* = 13 *n* (%)
Sex		
Female	21 (70%)	10 (77%)
Male	9 (30%)	3 (23%)
Marital status		
Single	1 (3.3%)	0 (0%)
Married	24 (80%)	13 (100%)
Divorced	4 (13.3%)	0 (0%)
Widow	1 (3.3%)	0 (0%)
Educational level		
Basic studies	23 (76.7%)	6 (46.1%)
Superior studies	7 (23.3%)	7 (53.9%)
Living area		
Rural	17 (56.7%)	7 (53.8%)
Urban	13 (43.4%)	6 (46.2%)
Confined at home		
Yes	26 (86.7%)	9 (69.2%)
No	4 (13.3%)	4 (30.8%)
Alone at home		
Yes	6 (20%)	0 (0%)
No	24 (80%)	13 (100%)
SOS Pain medication		
Yes	14 (46.7%)	6 (46.1%)
No	16 (53.3%)	0 (0%)
Not applied	0 (0%)	7 (53.8%)
Negative Impact from cancelled therapeutic massage		
Yes	10 (33.3%)	4 (30.8%)
No	6 (20%)	1 (7.7%)
Not applied	14 (46.7%)	8 (61.5%)
Contact with Pain Unit		
Yes	26 (86.7%)	7 (53.8%)
No	4 (13.3%)	6 (46.1%)
Adequate pain follow-up		
Yes	26 (86.7%)	6 (46.1%)
No	1 (3.3%)	0 (0%)
Maybe	3 (10%)	7 (53.8%)
Adequate healthcare follow-up		
Yes	26 (86.7%)	6 (46.1%)
No	1 (3.3%)	0 (0%)
I prefer not to respond	3 (10%)	7 (53.8%)
App as communication strategy		
Yes	24 (80%)	11 (84.6%)
No	2 (6.7%)	0 (0%)
Maybe	4 (13.3%)	2 (15.4%)
Strategies		
Frequent telephone calls	30 (100%)	13 (100%)
Videoconferences	12 (40%)	6 (46.2%)
Physical approaches (massage; acupuncture)	21 (70%)	4 (30.8%)
Psychosocial approaches (supportive therapy)	24 (80%)	7 (53.8%)

*n*—Sample size; %—Percentage.

**Table 2:** Depression, anxiety and stress assessment.

	Patients (*n* = 30)				Caregivers (*n* = 13)				Test U	*p*
	Middle Rank	SD	Min	Max	Middle Rank	SD	Min	Max		
Depression	21.74	3.60	7	23	20.96	4.39	7	20	181.50	0.85
Anxiety	22.48	3.88	7	22	17.42	5.23	7	22	131.00	0.23
Stress	21.80	3.70	7	20	20.75	4.83	7	22	171.00	0.82

SD—Standard Deviation; Min—Minimum value; Max—Maximum value; *n*—Sample size.

## Data Availability

The data are available upon reasonable request.

## Appendix A

**Table A1:** STROBE Statement—checklist of items that should be included in reports of observational studies.

	Item No	Recommendation	Page No
Title and abstract	1	(a) Indicate the study’s design with a commonly used term in the title or the abstract	1
		(b) Provide in the abstract an informative and balanced summary of what was performed and what was found	1
Introduction			
Background/rationale	2	Explain the scientific background and rationale for the investigation being reported	1–2
Objectives	3	State specific objectives, including any prespecified hypotheses	2
Methods			
Study design	4	Present key elements of study design early in the paper	3
Setting	5	Describe the setting, locations, and relevant dates, including periods of recruitment, exposure, follow-up, and data collection	3
Participants	6	(a) Give the eligibility criteria, and the sources and methods of selection of participants. Describe methods of follow-up	3
		(b) For matched studies, give matching criteria and number of exposed and unexposed	
Variables	7	Clearly define all outcomes, exposures, predictors, potential confounders, and effect modifiers. Give diagnostic criteria, if applicable	3–4
Data sources/measurement	8 *	For each variable of interest, give sources of data and details of methods of assessment (measurement). Describe comparability of assessment methods if there is more than one group	4
Bias	9	Describe any efforts to address potential sources of bias	4
Study size	10	Explain how the study size was arrived at	4–7
Quantitative variables	11	Explain how quantitative variables were handled in the analyses. If applicable, describe which groupings were chosen and why	4–7
Statistical methods	12	(a) Describe all statistical methods, including those used to control for confounding	
		(b) Describe any methods used to examine subgroups and interactions	4–7
		(c) Explain how missing data were addressed	
		(d) If applicable, explain how loss to follow-up was addressed	
		(e) Describe any sensitivity analyses	
Results			
Participants	13 *	(a) Report numbers of individuals at each stage of study—e.g., numbers potentially eligible, examined for eligibility, confirmed eligible, included in the study, completing follow-up, and analyzed	-
		(b) Give reasons for non-participation at each stage	
		(c) Consider use of a flow diagram	
Descriptive data	14 *	(a) Give characteristics of study participants (e.g., demographic, clinical, social) and information on exposures and potential confounders	4–7
		(b) Indicate number of participants with missing data for each variable of interest	-
		(c) Summarize follow-up time (e.g., average and total amount)	-
Outcome data	15 *	Report numbers of outcome events or summary measures over time	4–7
Main results	16	(a) Give unadjusted estimates and, if applicable, confounder-adjusted estimates and their precision (e.g., 95% confidence interval). Make clear which confounders were adjusted for and why they were included	
		(b) Report category boundaries when continuous variables were categorized	4–7
		(c) If relevant, consider translating estimates of relative risk into absolute risk for a meaningful time period	
Other analyses	17	Report other analyses performed—e.g., analyses of subgroups and interactions, and sensitivity analyses	-
Discussion			
Key results	18	Summarize key results with reference to study objectives	7
Limitations	19	Discuss limitations of the study, taking into account sources of potential bias or imprecision. Discuss both direction and magnitude of any potential bias	7
Interpretation	20	Give a cautious overall interpretation of results considering objectives, limitations, multiplicity of analyses, results from similar studies, and other relevant evidence	7
Generalizability	21	Discuss the generalizability (external validity) of the study results	7–8
Other information			
Funding	22	Give the source of funding and the role of the funders for the present study and, if applicable, for the original study on which the present article is based	9

* Give information separately for cases and controls in case–control studies and, if applicable, for exposed and unexposed groups in cohort and cross-sectional studies. Note: An Explanation and Elaboration article discusses each checklist item and gives methodological background and published examples of transparent reporting. The STROBE checklist is best used in conjunction with this article (freely available on the Web sites of PLoS Medicine at http://www.plosmedicine.org/ (accessed on 15 May 2023), Annals of Internal Medicine at http://www.annals.org/ (accessed on 15 May 2023), and Epidemiology at http://www.epidem.com/ (accessed on 15 May 2023)). Information on the STROBE Initiative is available at www.strobe-statement.org (accessed on 15 May 2023).
